# Extraction of natural antioxidants from Moroccan saffron (*Crocus sativus* L.) using ultrasound-assisted extraction: An optimization approach with box-behnken design

**DOI:** 10.1016/j.ultsonch.2025.107448

**Published:** 2025-06-26

**Authors:** Chaimae Slimani, Mouhcine Fadil, Chaimae Rais, Laila El-hanafi, Meryem Benjelloun, Hyeong-Moo Shin, John P. Giesy, Abderrahim Lazraq, Mahmoud M.A. Abulmeaty, Khalid M. Almutairi, Mourad A.M. Aboul-Soud

**Affiliations:** a Biotechnology, Environmental Technology and Valorization of Bio‑Resources Team, Department of Biology. Laboratory of Research and Development in Engineering Sciences Faculty of Sciences and Techniques Al‑Hoceima, Abdelmalek Essaadi University, Tetouan, Morocco; b Laboratory of Functional Ecology and Environmental Engineering, Sidi Mohamed Ben Abdellah University, Faculty of Sciences and Technologies, Department of Biology, P.O. Box 2202 – route d’Imouzzer, Fez, Morocco; c Laboratory of Botany, National Agency for Medicinal and Aromatic Plants, P.O. Box 159, Taounate, 34025, 10, Morocco; d Laboratory of Applied Organic Chemistry, Sidi Mohamed Ben Abdellah University, Faculty of Sciences and Technologies, Department of Biology, P.O. Box 2202 – route d’Imouzzer, Fez, Morocco; e Euromed University of Fez, UEMF, Morocco; f Department of Environmental Science, Baylor University, One Bear Place #97266, Waco, TX 76798, USA; g Department of Veterinary Biomedical Sciences and Toxicology Centre, University of Saskatchewan, Saskatoon S7N 5B3, Canada; h Department of Integrative Biology and Center for Integrative Toxicology, Michigan State University, East Lansing, MI 48823, USA; i Community Health Sciences Department, College of Applied Medical Sciences, King Saud University, Riyadh 11362, Kingdom of Saudi Arabia; j Center of Excellence in Biotechnology Research, College of Applied Medical Sciences, King Saud University, P.O., Riyadh 11433, Saudi Arabia

**Keywords:** Response Surface methodology, Box-Behnken design, Extraction parameters, Antioxidant activity, Natural products

## Abstract

There is increasing evidence of protective health effects of natural antioxidants, such as those contained in saffron (*Crocus sativus* L.), but it is challenging to extract polyphenols, which are the purported antioxidants with conventional techniques. This study developed an ultrasound-assisted extraction technique to improve the efficiency of phenolic compound extraction and assess the antioxidant activity of saffron. A response surface methodology, utilizing a Box-Behnken design, was employed to optimize three key extraction parameters: solid-to-liquid ratio, temperature, and extraction time for both saffron stigmas and corms. The total phenolic content and antioxidant activity were analyzed using the Folin-Ciocalteu and the 2,2-Diphenyl-1-picrylhydrazyl (DPPH) assays, respectively. The optimal extraction conditions for stigmas were a solid/liquid ratio of 40 mg/30 mL, with an extraction temperature of 50 °C for 15 min, resulting in a total phenolic content yield of 118.55 mg GAE/g DM and an IC_50_ value of 0.023 mg/mL. For optimal conditions when extracting from corms, the extraction time was extended to 45 min, resulting in a total phenolic content of 21.18 mg GAE/g DM and a IC_50_ value of 1.02 mg/mL. Results were consistent with predicted values provided by the quadratic model, which confirmed the efficacy of the Box-Behnken design in maximizing the extraction of polyphenol content from *C. sativus*. Although UAE has been refined at the laboratory level, additional research is needed to assess its feasibility and efficiency on an industrial scale. Exploring large-scale UAE systems, evaluating cost-effectiveness, and identifying potential adaptations for commercial production would be highly beneficial.

## 1 Introduction

Oxidative stress is a phenomenon characterized by an increase in the generation of free radicals within cells [1]. These radicals are naturally produced by the body, but they can become toxic if produced in excess [2]. Although this stress is not a disease itself, it is a significant contributor to or accessory factor in various health conditions. It can either initiate or exacerbate diseases like cancers, cardiovascular diseases, neurodegenerative disorders and diabetes [[3], [4], [5], [6], [7], [8]].

Saffron (*Crocus sativus*) is a perennial plant with distinctive anatomy. It has long, strap-shaped leaves that appear in the fall, followed by purple-violet flowers. The red stigma, known for its aromatic and medicinal qualities, is the most prized part of the plant. Below the surface, saffron has a corm, a bulbous structure that acts as the plant’s storage organ. The corm is thick, layered with a papery outer skin, and stores nutrients to help the plant survive dormant periods. It also supports the growth of new shoots during the flowering season and plays a vital role in the plant's annual regeneration [[9], [10], [11]].

Beyond its culinary appeal, saffron offers significant health benefits [[12], [13], [14], [15], [16]]. Saffron's abundant flavonoids and phenolic acids exhibit potential benefits for neurodegenerative disorders, diabetes management, cardiovascular wellness, and cancer prevention [[17], [18], [19], [20], [21]]. Their antioxidant and anti-inflammatory properties help combat oxidative stress, inhibit β-amyloid aggregation, and support metabolic regulation. Additionally, they improve endothelial function, modulate blood pressure, and exhibit anti-cancer effects by inducing apoptosis and inhibiting tumor growth, especially in synergy with crocin and safranal [[22], [23], [24]].

To date, conventional techniques like decoction, infusion, and Soxhlet extraction have limitations in the extractions of phenolic compounds from saffron, including oxidation, hydrolysis, ionization during extraction, and prolonged extraction times, affecting purity and yields of extracts [25,26]. In response to these challenges, alternative methods of extraction have been developed. Due to its cost-effectiveness, simplicity, and ability to match the efficiency of traditional methods, ultrasound-assisted extraction (UAE) is increasing in popularity [[27], [28], [29]].

UAE outperforms traditional methods in efficiency, sustainability, and preservation of phenolic compounds. Unlike maceration, which is slow and solvent-intensive, UAE accelerates extraction and enhances yield while using less solvent. Compared to Soxhlet extraction, UAE prevents heat-induced degradation and reduces energy consumption. While microwave speeds up extraction, it risks extracting unwanted compounds, whereas UAE offers better selectivity and compound stability. Overall, UAE provides the best balance of speed, efficiency, and bioactivity preservation, making it the superior method for phenolic extraction from saffron[[30], [31], [32]].

UAE uses high-frequency sound waves to enhance the extraction of bioactive compounds from plants. The ultrasound creates pressure changes that form microscopic bubbles, which collapse and generate shear forces, breaking down plant cell walls. This disruption releases compounds into the solvent more efficiently, leading to faster extraction, higher yields, and better quality. This process is also more sustainable, using less solvent, thus reducing both cost and environmental impact [[33], [34], [35]].

Studies on the extraction of polyphenol compounds from saffron using ultrasound-assisted techniques demonstrate diverse methodologies and outcomes that emphasize the optimization potential of this approach. [31] used a 50:50 methanol–water solvent for sonication, achieving a total phenolic content of 31.56 mg gallic acid equivalent (GAE) per gram of saffron extract. Similarly, results of previous studies [36] demonstrated that the ultrasound-assisted extraction produced a greater total phenolic content (103.063 ± 0.00 mg GAE/100 g) compared to conventional methods. These results demonstrate the efficiency gains of UAE over traditional extraction methods.

The efficiency of extraction of saffron depends on solvent type, solvent-to-solid ratio, time, temperature, and particle size. Solvent polarity affects solubility, while a higher solvent-to-solid ratio increases yields but might dilute the extract. The duration of extraction impacts interaction, with diminishing returns after equilibrium. Temperature enhances solubility but must be controlled to avoid degradation. Smaller particles improve efficiency but complicate filtration. For those reasons, optimal conditions are determined through studies and literature [30,37].

Achieving accurate evaluations of antioxidants from plants relies on optimizing extraction of constituents [[38], [39], [40]]. Results of some studies have confirmed the efficacy of applying a response surface methodology to assess and optimize extraction procedures by considering multiple parameters and their interactions [[41], [42], [43]].

The goal of this study was to optimize ultrasound-assisted extraction parameters to enhance the recovery of polyphenols and antioxidant activity from saffron (*Crocus sativus* L.) stigmas and corms. UAE was selected for its ability to improve extraction efficiency while preserving bioactive compounds. Using response surface methodology with a Box-Behnken design, key factors, including solid-to-liquid ratio, extraction temperature, and extraction time, all of which were optimized and their effects on total phenolic content and antioxidant activity evaluated. The findings offer valuable insights into the bioactive potential of saffron stigmas and corms for industrial applications.

## 2 Material and methods

### 2.1 Plant material

Plant materials utilized in this study were stigmas and corms of saffron, obtained from *C. sativus*, sourced from the Taliouine region in Taroudant province, Morocco. These botanicals were procured at the Fogoug Agricultural Cooperative from October to November 2019. Stigmas and corms were dried separately at 40 °C until dry mass was stabilized. Subsequently, the dried material was finely ground using an electric grinder, sieved to obtain particles of 300 μm size, and then stored in airtight vials at −28 °C until analysis.

### 2.2 Ultrasound-assisted extraction

Ultrasound at a steady frequency of 35 kHz was applied using an indirect ultrasonication bath in a hydro-ethanol solvent mixture (3:4 v/v), conducted in a darkened, cold room. The mixture was then centrifuged at 3,000 rpm for 10 min to separate the solid residue from the liquid extract. The resulting extract was stored at 4 °C in the dark until it was analyzed for its polyphenol content and antioxidant activity using 2,2-Diphenyl-1-picrylhydrazyl (DPPH). The analyses were conducted three times, and the data are presented as mean ± standard deviation (SD).

### 2.3 Total phenolic content determination

Total phenolic content (TPC) was quantified using a colorimetric assay based on the oxidation of phenols by Folin-Ciocalteu reagent and UV–vis spectrophotometer. A 200 µl of the extract was mixed with 1.5 mL of Folin-Ciocalteu reagent, which had been diluted 10 times with distilled water. After a 5-min interval, 1.5 mL of 5 % sodium carbonate solution was added. The absorbance was measured at 725 nm following 2 h of incubation in the dark. The resulting blue product is proportional to the phenol concentration [44]. A calibration curve with gallic acid was used to convert absorbance values to milligrams of gallic acid equivalents per gram of dry matter (mg GAE/g DM).

### 2.4 DPPH free radical-scavenging activity

To evaluate antioxidant capacity of saffron, samples were incubated with a DPPH solution. The extent of DPPH reduction, measured by the decrease in absorbance, determines the sample’s ability to scavenge free radicals [45]. A series of dilutions was prepared from a 4 mg/mL solution of each extract. Subsequently, 1 mL of each dilution was combined with 1 mL of 0.004 % DPPH solution. The mixture was left in dark for 30 min and the discoloration compared to the negative control containing only the DPPH solution was measured at 517 nm using UV–vis spectrophotometer. Effectiveness of these extracts as antioxidants was assessed by their capacity to scavenge 50 % of DPPH free radicals (IC_50_) (Eq. (1)) [46].

(1) Antioxidantactivity(%)=(AbsDPPH-Absfinal)/(AbsDPPH)×100

### 2.5 Statistical analysis

All statistical analyses were performed using JMP (v.14 pro) and Design Expert (v. 12) software. For optimizing extraction efficiency measured by both TPC and IC_50_, three key factors affecting extraction efficiency were identified as independent variables: material/solvent (X_1_, measured in mg/mL), temperature (X_2_, expressed as °C), and sonication time (X_3_, indicated in min). These three factors were chosen to ensure accurate adjustment for the extraction process. The Box-Behnken design employs three levels for each factor: lower (−1), middle (0), and upper (+1) levels. The actual values of these factors used at each level, as well as 15 randomly ordered combinations of these factors, are provided in Table 1. Note that central points were repeated three times. The Box-Behnken Design is favored for optimizing extraction parameters because it efficiently explores multiple factors with fewer experiments, saving time and cost. It ensures reliable results by testing all factor combinations without extremes, making it ideal for complex processes [47,48] (Table 2).

**Table 1:** Three extraction factors and their actual values used at each level and experimental design of ultrasonic-assisted extraction.

	Material/Solvent (mg/mL)	Temperature (°C)	Time (min)
Coded factors	X_1_	X_2_	X_3_
Lower (−1)	40/10	20	15
Middle (0)	40/20	35	30
Upper (+1)	40/30	50	45
Exp N°			
1	40/10	20	30
2	40/30	20	30
3	40/10	50	30
4	40/30	50	30
5	40/10	35	15
6	40/30	35	15
7	40/10	35	45
8	40/30	35	45
9	40/20	20	15
10	40/20	50	15
11	40/20	20	45
12	40/20	50	45
13	40/20	35	30
14	40/20	35	30
15	40/20	35	30

**Table 2:** Actual and predicted extraction results (TPC, IC_50_) from stigmas and corms of *C. sativus*.

N° Exp	Stigmas				Corms			
	TPC (mg GAE/ g DM)		IC_50_ (mg/mL)		TPC (mg GAE/ g DM)		IC_50_ (mg/mL)	
	Actual^a^	Predicted^b^	Actual^a^	Predicted^b^	Actual^a^	Predicted^b^	Actual^a^	Predicted^b^
1	82.13 ± 1.09	84.93	0.073 ± 0.0039	0.073	9.81 ± 1.12	8.84	2.56 ± 0.04	2.57
2	76.53 ± 2.20	77.36	0.025 ± 0.0038	0.025	18.46 ± 1.74	18.17	1.03 ± 0.0098	1.10
3	87.33 ± 2.16	86.50	0.066 ± 0.0013	0.066	16.96 ± 0.61	17.25	2.94 ± 0.0092	2.87
4	106.53 ± 1.89	103.73	0.022 ± 0.0096	0.022	16.41 ± 2.10	17.38	0.99 ± 0.0096	0.98
5	81.92 ± 3.25	81.78	0.068 ± 0.0069	0.068	16.79 ± 0.99	17.68	2.54 ± 0.0014	2.60
6	94.82 ± 2.24	96.65	0.024 ± 0.0027	0.024	17.82 ± 1.78	18.03	0.99 ± 0.004	0.99
7	96 ± 4.77	94.17	0.073 ± 0.0066	0.073	13.54 ± 0.89	13.33	2.71 ± 0.025	2.71
8	88.84 ± 2.25	88.98	0.024 ± 0.0073	0.024	23.33 ± 2.99	22.44	1.03 ± 0.026	0.97
9	76.66 ± 3.61	74.00	0.038 ± 0.0019	0.038	18.25 ± 1.28	18.33	1.49 ± 0.005	1.42
10	88 ± 0.73	88.97	0.031 ± 0.0047	0.031	24.33 ± 1.59	23.15	1.44 ± 0.063	1.45
11	78.33 ± 1.45	77.36	0.038 ± 0.0081	0.038	18.17 ± 0.33	19.36	1.43 ± 0.027	1.42
12	87.66 ± 1.17	90.32	0.034 ± 0.0065	0.034	22.25 ± 0.66	22.17	1.48 ± 0.006	1.55
13	84.66 ± 2.19	83.05	0.036 ± 0.0010	0.035	16 ± 0.96	16.36	1.4 ± 0.003	1.42
14	79.66 ± 1.17	83.05	0.034 ± 0.0072	0.035	16.75 ± 0.95	16.36	1.42 ± 0.009	1.42
15	84.83 ± 2.41	83.05	0.036 ± 0.0066	0.035	16.33 ± 0.87	16.36	1.45 ± 0.009	1.42

TPC: Total Phenolic Compounds; GAE: Gallic Acid equivalent; DM: Dry matter; a: observed values are the average of three replicates with standard deviation; b: predicted values from the final model.

Using the data in Table 1, the following polynomial model (full model) was first fit to each of the two responses (TPC and IC_50_) separately for stigmas and corms (Eq. (2)).

(2) $$Y\;=\;b_{0}\;+\;b_{1}X_{1}\;+\;b_{2}X_{2}\;+\;b_{3}X_{3}\;+\;b_{1}X_{1}X_{1}\;+\;b_{22}X_{2}X_{2}\;+\;b_{33}X_{3}X_{3}\;+\;b_{12}X_{1}X_{2}\;+\;b_{13}X_{1}X_{3}\;+\;b_{23}X_{2}X_{3}\;+\;\varepsilon$$

where Y is a response; b_0_ is an intercept or constant; b_1_, b_2_, and b_3_ are the coefficients of the main factors; b_11_, b_22_, and b_33_ are the coefficients of the quadratic terms; b_12_, b_13_, and b_23_ are the coefficients of the interaction terms; ε is error. Coefficients with *p-*values less than 0.05 were selected in the final (or reduced) models. Then, a residual analysis was performed to check the underlying assumptions of the errors and checked the model performance from the p-values of the lack-of-fit test and the coefficient of determination (R^2^).

## 3 Results

### 3.1 Effect of extraction parameters on polyphenol content and antioxidant activity

Total polyphenol content (TPC) varied as a function of the combinations of the three selected extraction parameters (Table 3). Among the 15 experiments, experiment number 4 (40 mg/30 mL ratio; 50 °C and 30 min of extraction) provided the greatest yield of phenolic compounds (106.53 mg GAE/g DM) for stigmas. The set of these same parameters recorded the best IC_50_ for stigmas (0.022 mg/mL). In contrast, experiment number 2 (40 mg/30 mL ratio; 20 °C and 30 min of extraction) produced the least TPC, while experiment number 1 (40 mg/10 mL ratio; 20 °C and 30 min of extraction) had the greatest IC_50_.

**Table 3:** Lack-of-fit statistics for two responses (TPC and IC_50_) of extracts of stigmas and corms of *C. sativus*.

ANOVA source	DF	TPC				IC_50_			
		SS	MS	F	*p-*value	SS	SM	F	*p-*value
Stigmas									
Regression	9	868.48	96.49	8.4508	0.0150*	0.00485632	0.000540	789.645	0<.0001*
Residual	5	57.09	11.41			0.00000342	6.833e-7		
Lack of fit	3	39.841675	13.2806	1.5395	0.4171	0.00000075	2.5e-7	0.1875	0.8972
Pure Error	2	17.252600	8.6263			2.6666e-6	1.333e-6		
R^2^	0.938					0.999			
Corms									
Regression	9	178.88270	19.8759	14.5081	0.0044*	6.3186583	0.702073	116.422	0<.0001*
Residual	5	6.84990	1.3700			0.0301417	0.006028		
Lack of fit	3	6.5673000	2.18910	15.4926	0.0612	0.02887500	0.009625	15.1974	0.0624
Pure Error	2	0.2826000	0.14130			0.00126667	0.000633		
R^2^	0.963					0.995			

TPC: Total Polyphenol Content; DF: degrees of freedom; SS: sum of squares; MS: mean square; R: regression; r: residual; *: statistically significant at the level of p < 0.05.

For the corm extracts, the largest TPC value (24.33 mg GAE/g DM) was obtained in experiment 10, using a 40 mg/20 mL ratio, 50 °C, and 15 min of extraction. Additionally, the antioxidant capacity, indicated by the IC50 value, varied among extracts. The optimum IC50 value was 0.99 mg/mL. Experiment 1, on the other hand, showed the minimum TPC (9.81 ± 1.12 mg GAE/g DM) and the greatest IC_50_ (2.56 ± 0.04 mg/mL).

The absence of correlation between TPC and IC_50_ in saffron corm extracts can be attributed to the differing bioactivity of phenolic compounds, their structural variations, and interactions among phytochemicals. To assess antioxidant potential, both the quantity and quality of phenolics should be considered, along with thorough bioactivity testing.

Lack-of-fit statistics (Table 3) indicated no lack of fit at the 5 % level for two responses (TPC and IC_50_) of both stigmas and corms (*p-*values for LF > 0.05). Non-significant lack of fit indicates that the model fits well and provides adequate predictability for the responses. In addition, large R^2^ is good for fitting our responses. This good concordance was confirmed by the graph of observed results against predicted ones (Fig. A.1).

### 3.2 Effects of extraction parameters

The effects of three extraction parameters, encompassing material/solvent ratio (X_1_), temperature (X_2_), and time (X_3_) as well as its quadratic terms and interaction terms among parameters, were investigated, during use of the ultrasound extraction method. Overall, results are similar between stigmas and corms (Table 4). Positive coefficients in interaction terms indicate that two factors work together to increase responses, while negative coefficients indicate that they work together to decrease responses [49].

**Table 4:** Regression coefficients for total polyphenol content (TPC) & IC_50_ for antioxidant of *C. sativus* and their level of significance.

		Stigmas				Corms			
Terms	Coefficient	TPC		IC_50_		TPC		IC_50_	
		Estimate	*p-value*	Estimate	*p-value*	Estimate	*p-value*	Estimate	*p-value*
Intercept	b_0_	83.05	0<.0001*	0.035	0<.0001*	16.36	0<.0001*	1.42	0<.0001*
Solvent to material ratio	b_1_	2.41	0.0989	−0.023	0<.0001*	2.365	0.0023*	−0.83	0<.0001*
Temperature	b_2_	6.98	0.0021*	−0.002	0.0003*	1.907	0.0058*	0.042	0.1822
Time	b_3_	1.17	0.3691	0.001	0.0188*	0.012	0.9771	0.023	0.4265
Solvent to material ratio × Temperature	b_12_	6.2	0.0145*	0.001	0.0602	−2.3	0.0111*	−0.10	0.0425*
Solvent to material ratio × Time	b_13_	−5.01	0.0312*	−0.001	0.0293*	2.19	0.0134*	−0.032	0.4407
Temperature × Time	b_23_	−0.50	0.7781	0.0007	0.1293	−0.5	0.4319	0.025	0.5480
Solvent to material ratio × Solvent to material ratio	b_11_	6.40	0.0149*	0.011	0<.0001*	−1.915	0.0256*	0.40	0.0002*
Temperature × Temperature	b_22_	−1.32	0.4847	−0.0004	0.3773	0.965	0.1740	0.049	0.2744
Time × Time	b_33_	0.93	0.6163	0.0003	0.4735	3.425	0.0025*	−0.012	0.7621

*: statistically significant at 0.05

TPC: Total polyphenol content

For extracts of stigmas of saffron, the response TPC was influenced by significant terms (*p-*values < 0.05), including the constant term (b_0_), temperature (b_2_), the interaction terms of b_12_ and b_13_, and the quadratic terms of b_11_. The resulting relationship between response and factors formed a second-order polynomial equation (Eq. (3)). Similar relationships were observed for IC_50_ of stigmas (Eq. (4)), TPC of corms (Eq. (5)), and IC_50_ of corms (Eq. (6)).

(3) $$Y\;=\;83.05\;+\;6.98X_{2}\;+6.2X_{1}X_{2}\;-\;5.015X_{1}X_{3}\;+\;6.4X_{1}X_{1}\;+\;\varepsilon$$

(4) $$Y\;=\;0.035\;-0.023\;X_{1}\;-0.002X_{2}\;+0.001X_{3}\;-\;0.001X_{1}X_{3}\;+\;0.011X_{1}X_{1}\;+\;\varepsilon$$

(5) $$Y\;=\;16.36\;+\;2.365X_{1}\;+1.907X_{2}\;-\;2.3\;X_{1}X_{2}\;+\;2.19X_{1}X_{3}\;-1.915X_{1}X_{1}+\;3.425X_{3}X_{3}\;+\;\varepsilon$$

(6) $$Y\;=\;1.42\;-\;0.83X_{1}-\;0.1\;X_{1}X_{2}\;+0.4\;X_{1}X_{1}\;+\;\varepsilon$$

### 3.3 Optimization and desirability of parameters

**Fig. 1:**
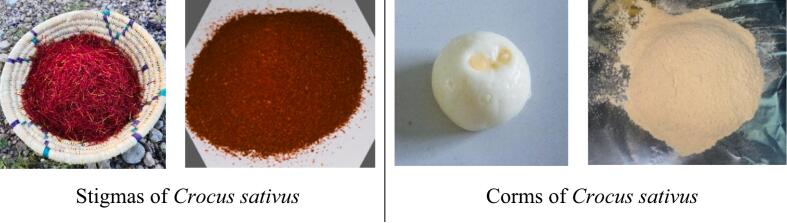
*Crocus sativus* stigmas and corms utilized in this study.

A contour plot based on *iso*-response curves was employed to assess the quality of the postulated models and attain optimal values for the four responses. A preliminary analysis indicates that minimizing the duration of sonication to its minimum is crucial for optimizing stigmas responses, while maximizing the duration of sonication was more recommended for corms. Given that the *iso*-response profile is a 2D representation, this factor was fixed at its minimum duration (15 min) for stigmas and longer for corms (Fig. 1). This step is crucial to isolate and assess the influence of the other two factors by plotting the profile.

#### 3.3.1 Adequacy of the extraction parameters on TPC and IC_50_ of extracts of stigmas

**Fig. 2:**
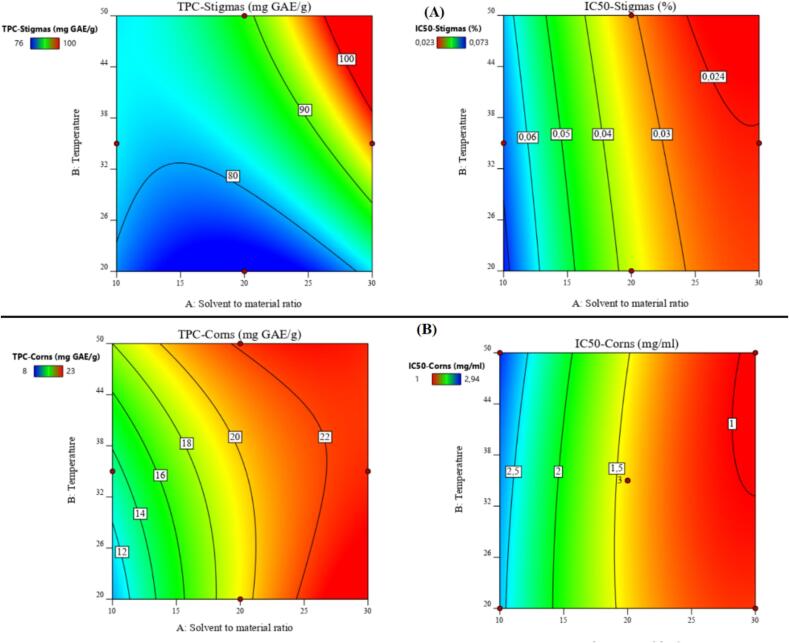
2-D representations showing the combined effect of parameters from the Box- Benhken design. Influence of extraction parameters on total polyphenol content (TPC) and antioxidant activity (IC_50_) in the extract from (A) stigmas and (B) corms.

The relationship between extraction parameters and the content of phenolic compounds and antioxidant activity was further studied using response surface diagrams. For extracts of stigmas, TPC exceeding 100 mg GAE/g (red zone) and an IC_50_ of approximately 0.024 mg/mL can be achieved by fixing the sonication time at its minimum value (15 min), maintaining temperature between 38 and 50 °C, and utilizing a material/solvent ratio ranging from 40 mg/25 ml to 40 mg/30 mL (Fig. 2A). Additionally, the desirability function indicates a 99 % probability of reaching values of 109.09 mg GAE/g DM for TPC and 0.021 mg/mL for IC_50_ by setting temperature to 50 °C, the material/solvent ratio at 40 mg/30 ml, and the sonication time at 15 min (Fig. A2).

#### 3.3.2 Adequacy of the extraction parameters on TPC and IC_50_ of extracts of corms

TPC exceeding 22 mg GAE/g DM (red zone) and an IC_50_ of approximately 1 mg/mL can be achieved by fixing the sonication time at its maximum value (45 min) and ensuring maximal temperature and material/solvent ratio (Fig. 2B). Furthermore, the desirability function suggests a 99 % likelihood of attaining values of 22.5 mg GAE/g for TPC and 0.98 mg/ml for IC_50_ by maintaining the temperature at 50 °C, the material/solvent ratio at 40 mg/30 mL, and the sonication time at 45 min (Fig. A2).

### 3.4 Experimental validation of optimal conditions

Empirical values were comparable with the predicted values from the final selected model, and there was no significant difference between experimental and predicted responses (Table 5). Therefore, we can conclude that the three extraction factors optimized from a response surface methodology are reliable enough to predict polyphenol yield and antioxidant activity of extracts.

**Table 5:** Predicted values of TPC and IC_50_ from the final selected model and those observed in our optimal experiment.

	Three extraction factors			TPC (mg GAE/ g DM)		IC_50_ (mg/mL)	
	Material/ solution (mg/mL)	Temperature (°C)	Time (min)	Predicted values^a^	Observed values^b^	Predicted values^a^	Observed values^b^
Stigmas	40/30	50	15	109.00 ± 10.27	118.55 ± 0.13	0.021 ± 0.0025	0.023 ± 0.0002
Corms	40/30	50	45	22.51 ± 3.55	21.18 ± 0.90	0.98 ± 0.23	1.02 ± 0.01

a: The predicted value is given with the standard deviation of the response calculated from the model.

b: The observed value is the average of three replicates with standard deviation.

## 4 Discussion

Three factors, including the material/solvent ratio, temperature, and time, are crucial in extracting the phenolic compounds from plant matrices. That is, the extraction conditions of these factors could exert notable impact on the antioxidant activity of the extract [41,[50], [51], [52], [53]]. The results of this study identified optimal conditions that improve the yield of total phenols and consequently enhance the antioxidant activity of the saffron plant by employing response surface methodology and Box-Behnken design, with a goal of maximizing the desired responses.

This study demonstrated that the material/solvent ratio was the most influential factor affecting the yield of polyphenols and antioxidant activity of saffron extracts. Having an excess of solvent can result in dilution of target compounds, causing a reduction in their concentration within the extraction mixture. Conversely, insufficient solvent can fail to interact adequately with the sample, affecting the kinetics of the extraction process. This can result in lesser efficiencies of extraction and a need for greater durations of extraction. Thus, ensuring an optimal plant material-to-solvent ratio is essential for achieving efficient extraction, as demonstrated by two studies [54,55]. Similarly, studies on *C. sativus* have utilized response surface methodology to optimize extraction parameters for polyphenols, revealing that a lesser material-to-solvent ratio leads to the highest polyphenol yield [50,56,57].

Temperature is also an important factor in phenol extraction, as demonstrated previously [[58], [59], [60], [61], [62]]. This finding is consistent with the conclusions drawn from the study discussed here, indicating that increasing temperatures significantly enhanced the yield of phenolic compounds from *C. sativus*. While higher temperatures improve solubilities of phenolic, it also affects the hydrolysis reaction of the cell wall due to the increase in the ionization constant [63]. In addition, higher temperatures soften plant tissue, disrupting interactions between polyphenol and proteins. This increases polyphenol solubility and enhances extraction efficiency [64,65]. Furthermore, higher temperatures reduce solvent surface tension and viscosity, facilitating better penetration into the plant matrix. This enhances diffusion and molecular transfer rates during extraction [[66], [67], [68], [69]]. At 50 °C, extraction polyphenol was maximum from both *C. sativus* stigmas and corms. However, temperatures greater than 60 °C can degrade thermosensitive polyphenol content, aligning with previous studies [9,70]. The results of this experiment also showed a negative quadratic effect of temperature (X_2_) on the desired response, emphasizing the need for careful optimization of extraction temperature based on target content.

Optimal duration of extraction is the shortest possible time to extract a maximum of the phenols and establish an equilibrium between the solutes of the plant matrix and the solvent [71,72]. In this study, a duration of 15 min was sufficient to extract a maximum of the polyphenols from the stigmas of *C. sativus*. However, keeping the temperature at 50 °C, the corms need more time, about 45 min to reach the optimal response. Extended exposure of corms in solvent enables desired compounds to migrate. Some reports have recommend 40 min of ultrasound for optimal phenol extraction from saffron by-products, ensuring extracts with maximum antioxidant activity and phenolic content [70,73].

The interaction between material/solvent ratio and temperature is crucial for extracting phenolic compounds efficiently. Higher temperatures enhance solvent properties, improving diffusion and solubility, but an unoptimized material/solvent ratio can hinder solvent penetration, reducing yields. Additionally, heat-sensitive compounds may degrade at high temperatures, affecting extract quality [[74], [75], [76]]. A higher material/solvent ratio can increase solute concentration, aiding diffusion, but too short an extraction time may prevent optimal solvent penetration, while excessive time can lead to degradation or leaching of unwanted compounds [43,77]. Therefore, optimizing both the material/solvent ratio and extraction time is essential for maximizing yield and preserving bioactivity [78]. The relationship between extraction time and temperature is also significant; higher temperatures generally promote the solubility of phenolic compounds, but prolonged exposure can cause degradation. Understanding these dynamics emphasizes the need to balance extraction parameters to achieve the best results [79,80].

The study conducted by [81] on saffron stigmas from Taliouine, Morocco, reported a polyphenol concentration of approximately 56.11 ± 4.75 mg GAE/g DM and an IC_50_ value of 1.7 ± 0.023 mg/mL using the maceration extraction technique. Similarly, [82] found a higher concentration of 97.99 mg GAE/g in Algerian saffron stigmas. [83] recorded a yield of 45.47 mg GAE/g DM using an ethanol–water extraction method on stigmas from the Boulemane region, Morocco.

An ultrasound-assisted extraction combined with response surface methodology (RSM) was applied and the results demonstrated total phenolic content of 103.063 ± 0.00 mg GAE/100 g [36]. Additionally, [84] reported an IC_50_ value of 38.99 mg/mL. Also, [85] optimized the extraction of bioactive compounds from *Crocus sativus* corms in Iran using response surface methodology (RSM) and a central composite design (CCD) combined with ultrasound-assisted extraction. Their study reported a yield of 100.39 mg GAE per 100 g of dry matter (DM).

Compared to previous studies, the findings of the study, results of which are presented here, indicate a polyphenol concentration of 118.55 mg GAE/g DM and a IC_50_ value of 0.023 mg/mL for saffron stigmas, while saffron corms exhibited a polyphenol content of 21.18 mg GAE/g DM and an IC_50_ of 1.02 mg/mL. These results demonstrate superior polyphenol amounts and strong antioxidant capacity of *Crocus sativus* stigmas from the Taliouine region, emphasizing their potential biological and pharmacological benefits.

To maximize the benefits of optimized extraction conditions for polyphenols from saffron, several recommendations emerge from recent studies. First, establish standardized extraction protocols that use optimized parameters, including solvent composition, temperature, and sonication time. For example, our study identified that a 40 mg/30 mL solvent ratio, 50 °C temperature, and sonication times of 15 min for stigmas and 45 min for corms yield higher total phenolic content.

The differing extraction times for saffron stigmas and corms can be attributed to their structural differences. Corms have thicker, lignified cell walls that hinder solvent diffusion and phenolic compound release, requiring longer extraction periods. In contrast, the looser structure of stigmas allows for quicker solvent penetration. Additionally, the density and composition of plant tissues affect extraction efficiency, with denser corm tissues needing more time for effective solvent interaction. Even under constant temperature, diffusion rates can vary significantly, influencing extraction outcomes. The specific interactions between solvents and phenolic compounds may also necessitate longer extraction times for complete dissolution and interaction [86,87].

Employing response surface methodology (RSM) can systematically analyze interactions between extraction variables, helping to determine optimal conditions that enhance polyphenol yield with efficient resource use. Finally, these extracted polyphenols can be used as natural preservatives or antioxidants in food products, potentially replacing synthetic additives. The strong antioxidant activity found in optimized extracts (e.g., IC_50_ values of 0.023 mg/mL and 1.02 mg/mL for stigmas and corms, respectively) indicates their suitability for improving food quality and shelf life. The corms of saffron, often considered waste, contain significant amounts of bioactive compounds.

The results of this study demonstrate significant differences in total polyphenol content and IC_50_ values between saffron stigmas and corms. Stigmas, as reproductive structures, are likely to contain higher levels of flavonoids and carotenoids, while corms may have more phenolic acids and tannins, reflecting their distinct physiological roles [88]. The synthesis pathways for these compounds vary, with stigmas potentially producing more phenolics due to environmental stress, while corms accumulate compounds for resilience against pathogens[89,90]. Additionally, structural differences, such as thicker cell walls in corms, can affect extraction efficiency, influencing TPC and IC_50_ [91].

Optimizing extraction parameters for practical applications involves balancing trade-offs between time and energy costs. While longer extraction times can increase yields of phenolic compounds, they may reduce operational efficiency in industrial processes. Shorter extraction times enable faster turnaround but often yield less, affecting profitability. Energy-efficient methods like ultrasonic-assisted extraction (UAE) can lower energy costs while enhancing yield. Innovations in equipment and techniques can improve efficiency without significantly raising energy usage. Finding the right balance is crucial to maximizing yield while maintaining the quality and bioactivity of the extracted compounds. Hence, it can be inferred that the stigmas and by-products of *C. sativus* possess bioactive compounds with the potential for utilization in valuable products. These could find applications in diverse fields, such as pharmaceuticals, cosmetics, food, and medicine.

## 5 Conclusions

Saffron plants have antioxidant-rich phenolic compounds. This study explored how extraction conditions, like material/solvent ratio, temperature, and duration of extraction, affect phenolic yield and scavenging activity using response surface methodology and the Box-Behnken design. The ideal extraction conditions for stigmas were a solid/liquid ratio of 40 mg/30 mL, with an extraction temperature of 50 °C for 15 min, yielding a total phenolic content of 118.55 mg GAE/g DM and an IC_50_ value of 0.023 mg/mL. For corm extraction, the optimal conditions involved extending the extraction time to 45 min, resulting in a total phenolic content of 21.18 mg GAE/g DM and an IC_50_ value of 1.02 mg/mL. These results represent a significant advancement in the optimization of polyphenol content extraction and thus could indicate notable progress in effectively controlling the extraction of phenolic compounds for determined objectives.

## CRediT authorship contribution statement

**Chaimae Slimani:** Writing – original draft, Methodology, Investigation, Data curation. **Mouhcine Fadil:** Writing – review & editing, Supervision, Methodology, Data curation. **Chaimae Rais:** Writing – review & editing, Supervision, Methodology. **Laila El-hanafi:** Writing – review & editing. **Meryem Benjelloun:** Writing – review & editing, Validation. **Hyeong-Moo Shin:** Writing – review & editing, Methodology, Formal analysis. **John P. Giesy:** Writing – review & editing, Supervision. **Abderrahim Lazraq:** Validation. **Mahmoud M.A. Abulmeaty:** Writing – review & editing, Validation. **Khalid M. Almutairi:** Writing – review & editing, Visualization, Validation, Investigation, Funding acquisition. **Mourad A.M. Aboul-Soud:** Writing – review & editing, Visualization, Validation, Investigation.

## Funding

The authors would like to thank Ongoing Research Funding Program, (ORFFT-2025-002-1), King Saud University, Riyadh, Saudi Arabia for financial support.

## Declaration of competing interest

The authors declare that they have no known competing financial interests or personal relationships that could have appeared to influence the work reported in this paper.
